# Biological significance of METTL5 in atherosclerosis: comprehensive analysis of single-cell and bulk RNA sequencing data

**DOI:** 10.18632/aging.205755

**Published:** 2024-04-24

**Authors:** Jianjin Wu, Lei Wang, Shuaishuai Xi, Chao Ma, Fukang Zou, Guanyu Fang, Fangbing Liu, Xiaokai Wang, Lefeng Qu

**Affiliations:** 1 Department of Vascular and Endovascular Surgery, Second Affiliated Hospital of Naval Medical University, Shanghai, China; 2 Department of Vascular Surgery, First Affiliated Hospital of Dalian Medical University, Dalian 116011, China; 3 Department of Vascular Surgery, Weifang Yidu Central Hospital, Weifang, Shandong, China; 4 Department of Interventional and Vascular Surgery, The First People’s Hospital of Xuzhou, Xuzhou, Jiangsu, China

**Keywords:** atherosclerosis, m6A, single-cell, immune infiltration, WGCNA

## Abstract

Background: N6-methyladenosine (m6A) methylation is involved in the pathogenesis of atherosclerosis (AS). Limited studies have examined the role of the m6A methyltransferase METTL5 in AS pathogenesis.

Methods: This study subjected the AS dataset to differential analysis and weighted gene co-expression network analysis to identify m6A methylation-associated differentially expressed genes (DEGs). Next, the m6A methylation-related DEGs were subjected to consensus clustering to categorize AS samples into distinct m6A subtypes. Single-cell RNA sequencing (scRNA-seq) analysis was performed to investigate the proportions of each cell type in AS and adjacent healthy tissues and the expression levels of key m6A regulators. The mRNA expression levels of METTL5 in AS and healthy tissues were determined using quantitative real-time polymerase chain reaction (qRT-PCR) analysis.

Results: AS samples were classified into two subtypes based on a five-m6A regulator-based model. scRNA-seq analysis revealed that the proportions of T cells, monocytes, and macrophages in AS tissues were significantly higher than those in healthy tissues. Additionally, the levels of m6A methylation were significantly different between AS and healthy tissues. METTL5 expression was upregulated in macrophages, smooth muscle cells (SMCs), and endothelial cells (ECs). qRT-PCR analysis demonstrated that the METTL5 mRNA level in AS tissues was downregulated when compared with that in healthy tissues.

Conclusions: METTL5 is a potential diagnostic marker for AS subtypes. Macrophages, SMCs, and ECs, which exhibit METTL5 upregulation, may modulate AS progression by regulating m6A methylation levels.

## INTRODUCTION

Atherosclerosis (AS), a cardiovascular disease (CVD), is characterized by the thickening and hardening of arterial walls due to the accumulation of cells, cholesterol, and extracellular matrix components [1–4]. Epidemiological studies have revealed that high blood pressure, tobacco use, diabetes, and high cholesterol levels are the risk factors for AS and related pathological processes [1, 5, 6]. Recent studies have demonstrated that the combination of aging and inflammation increases the risk of developing AS [7, 8]. Advances in the medical field have improved AS treatment outcomes but have not markedly alleviated the health risks and societal burden. The development of AS is associated with cellular and molecular changes, such as epigenetic modifications [9, 10]. Hence, novel molecular targets must be identified to enable early detection, risk evaluation, and targeted therapy development for AS.

In mammalian cells, N6-methyladenosine (m6A) is the predominant RNA chemical modification [11, 12]. m6A methylation is catalyzed by m6A methyltransferase (writer), removed by m6A demethylase (eraser), and read by RNA-binding proteins (reader). Recent studies have indicated that m6A modification is associated with the pathogenesis of various diseases, including CVDs and cancer [11, 13, 14]. However, the biological function of METTL5 has not been previously reported in AS.

This study performed differential analysis and weighted gene co-expression network analysis (WGCNA) to identify m6A-related differentially expressed genes (DEGs). Next, consensus clustering was used to classify AS samples into two m6A subtypes. The cell-type identification by estimating relative subsets of RNA transcripts (CIBERSORT) algorithm was used to calculate the immune infiltration scores for each AS sample and evaluate the differential infiltration levels of immune cells between the m6A subtypes. The functions of DEGs between the two m6A subtypes were determined using functional enrichment analysis. The proportions of different cell types in AS and healthy tissues and the expression levels of key m6A regulators were determined using single-cell RNA sequencing (scRNA-seq). Finally, a nomogram based on m6A-related genes (M6ARGs) was developed to evaluate the risk of developing AS.

## METHODS

### Data acquisition and pre-processing

The GSE100927 dataset, which comprises the data of 35 healthy individuals and 69 patients with AS, was downloaded from the Gene Expression Omnibus (GEO) database (http://www.ncbi.nlm.nih.gov/geo). The Perl script and the R package “limma” were used to process and normalize the expression profiles of the GSE100927 dataset. From previous studies, 40 M6ARGs were retrieved.

### Processing of scRNA-seq data

The scRNA-seq dataset GSE159677 was retrieved from the GEO database. The dataset comprises scRNA-seq data of 3 carotid artery plaques and 3 adjacent healthy tissues. Batch correction in the samples was performed using the ‘Seurat’ software package. The data of cells with a gene count of 200–6000 and a mitochondrial gene proportion of <25% were included, whereas those of cells with low-quality data were excluded from the dataset. The data were standardized using the “Normalize Data” function. Genes with specific expression patterns were identified using the “FindVariableFeatures” function. The “RunPCA” function was used for clustering and uniform manifold approximation and projection, a manifold learning technique for dimension reduction [15, 16]. The “FindMarkers” function was used to analyze DEGs in various cell subtypes. Genes specific to each cell cluster were identified, and cells were annotated using the “SingleR” R package. The “VlnPlot” function from the “Seurat” package was used to generate violin plots depicting the differential m6A scores and m6A regulators between cell types.

### WGCNA

The co-expression modules were identified using WGCNA with the R package ‘WGCNA’ [17]. To generate a topological overlap matrix (TOM), the ideal soft threshold power was determined to generate a weighted adjacency matrix. Next, the TOM dissimilarity measure (1-TOM) was used to generate modules using the hierarchical clustering tree algorithm, ensuring a minimum module size of 60. A random color was assigned to each module. The eigengene module was used to represent the overall gene expression profiles in each module. To determine the correlation between modules and disease status, the module significance values were calculated. “Gene significance” was used to establish the correlation between a gene and a clinical trait.

### Random forest (RF) and support vector machine (SVM)

The RF algorithm, a powerful and widely applicable machine learning algorithm, is used for classification and regression analysis [18]. SVM is a type of generalized linear classifier that operates in a supervised learning manner for binary classification of data [19]. AS-associated m6A regulators were screened using the RF [18] and SVM-Recursive Feature Elimination (RFE) [19] algorithms. The RF classifier was generated using the R package “Random Forest,” while the SVM classifier was generated using the R package “e1071”.

### Immune landscape analysis

The CIBERSORT algorithm was used to determine the relative abundances and infiltration scores of 22 immune cell types in each sample [20]. The correlation between five m6A regulators and the infiltration levels of 22 immune cell types was examined. The results were presented using the R packages “reshape2” and “ggpubr”.

### Consensus clustering

Based on the expression levels of five m6A regulators, the AS samples were categorized into different m6A subtypes using the R package “Consensus Cluster Plus”. Based on the consensus matrix and cumulative distribution function (CDF), the maximum cluster number was determined to be 9, which was chosen as the optimal number of clusters. Principal component analysis (PCA) was performed to further examine the distribution among the clusters associated with m6A. Furthermore, the differential infiltration levels of immune cells between various m6A clusters were examined.

### Gene set variation analysis (GSVA)

GSVA enables the evaluation of the relative enrichment levels of gene sets in samples. This approach assesses the activity of gene sets in different samples, revealing differential biological processes, cellular functions, or pathways. The differential biological activities between the two m6A subtypes were determined using the “GSVA” R package. The GSVA gene sets were obtained from the modules of the MSigDB database called “curated gene sets” and “ontology gene sets”.

### Gene ontology (GO), Kyoto encyclopedia of genes and genomes (KEGG), and disease ontology (DO) analyses

The biological functions of the DEGs between the m6A subtypes were assessed using functional enrichment analysis. GO enrichment analysis determines the enrichment of genes in molecular functions, biological processes, and cellular components. Meanwhile, KEGG pathway enrichment analysis determines the pathways in which DEGs are enriched. Disease Ontology (DO) enrichment analysis was performed to examine the correlation between the disease and DEGs.

### Construction of a nomogram and the receiver operating characteristic (ROC) curves

Based on the expression levels of five m6A regulators, a nomogram model based on M6ARGs was constructed to predict the risk of developing AS. The prediction accuracy of the nomogram was validated using decision curve analysis (DCA) and a calibration curve. The diagnostic efficacy of five m6A regulators in AS subtypes was examined using ROC curve analysis.

### Quantitative real-time polymerase chain reaction (qRT-PCR)

Total RNA was extracted from AS and adjacent healthy tissues using Trizol reagent (Thermo Fisher Scientific) and reverse-transcribed into complementary DNA, following the manufacturer’s instructions, to examine the m6A regulator expression levels. qRT-PCR analysis was performed using the SYBR Green master kit (Vazyme) with a LightCycler 480 II (Roche Diagnostics) instrument. The following primers were used for amplifying METTL5: 5′-GGGTTAGCCGGGAGATCCT-3′ (forward) and 5′-GCAGGCGACTCTCTAGTTCC-3′ (reverse).

### Statistical analysis

The differential analysis between AS tissues and normal tissues was performed using R software (Version 4.2.1). Differences were considered significant at *P* < 0.05.

### Availability of data and materials

The datasets presented in this study can be found in online repositories. The names of the repository/repositories and accession number(s) can be found in the article.

## RESULTS

### WGCNA results

**Figure 1:**
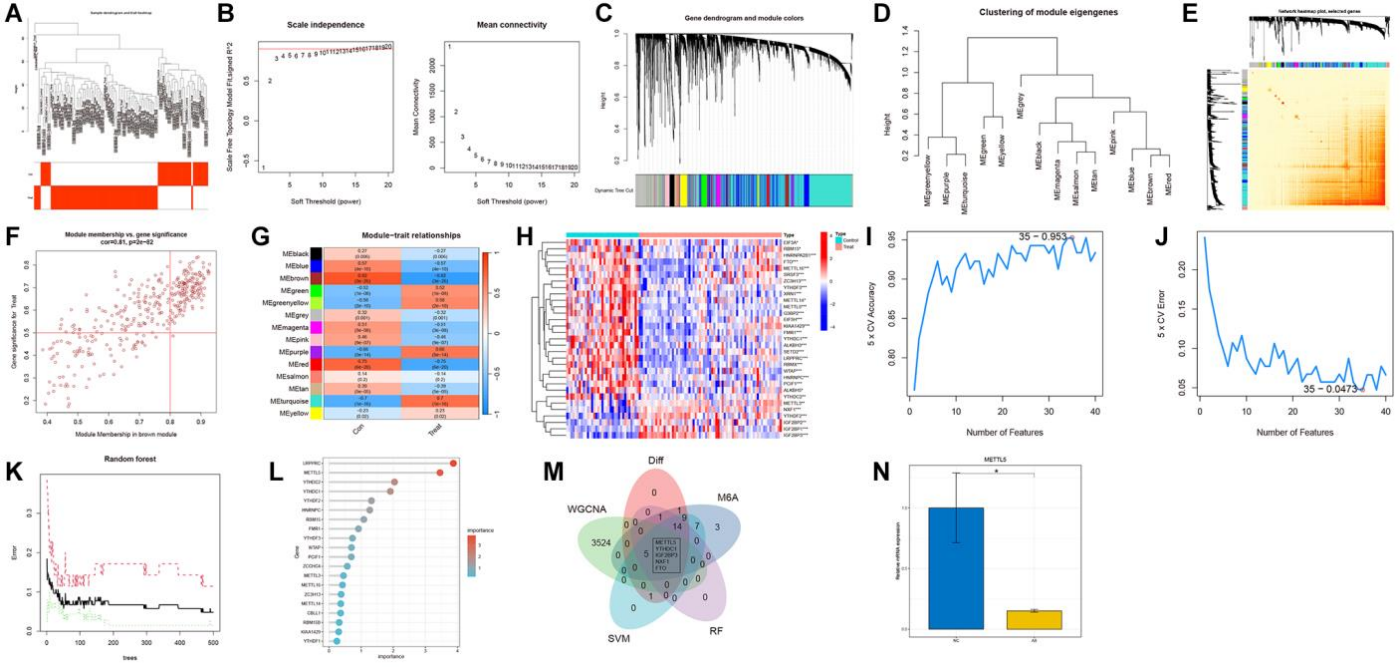
**Construction of the co-expression network using weighted gene co-expression network analysis (WGCNA).** (**A**) Sample clustering dendrogram with tree leaves corresponding to individual samples. (**B**) Soft threshold β = 10 and scale-free topological fit index (R2). (**C**) The original and combined modules of the clustering tree. (**D**) Representative clustering of module eigengenes. (**E**) Representative heatmap of the correlation between 14 modules. (**F**) Scatter plot between module membership in brown module and the gene significance for AS. (**G**) Correlation analysis between module eigengenes and clinical status. Each row represents a module, while each column represents a clinical status. (**H**) The expression patterns of 31 N6-methyladenosine (m6A)-related genes (M6ARGs) were presented in the heatmap. (**I**, **J**) Biomarker signature gene expression validation using the support vector machine recursive feature elimination (SVM-RFE) algorithm. (**K**) Random Forest error rate versus the number of classification trees. (**L**) The top 20 relatively important genes. (**M**) Venn diagram for screening genes. (**N**) Relative mRNA level of METTL5 in atherosclerosis (AS) (yellow bars) and healthy tissues (blue bars). ^*^p < 0.05, ^**^p < 0.01 and ^***^p < 0.001.

To identify AS-related gene modules, co-expression networks and modules were constructed for the control and treatment groups using WGCNA. A scale-free network was constructed using β = 10 as the soft threshold (Figure 1A, 1B). Subsequently, 7638 genes were divided into 14 distinct modules with different colors (Figure 1C–1E). As shown in Figure 1F, the brown module was strongly associated with AS (R = 0.82, *P* = 3e-26). Finally, 3529 hub genes were identified from this module based on specific criteria (Figure 1G).

### DEG screening

The “limma” package was used to examine 31 M6ARGs (6 upregulated genes and 25 downregulated genes) (Figure 1H). The SVM-RFE machine learning algorithm was used to identify 35 M6ARGs (Figure 1I, 1J). The combination of RF and feature selection was used to establish the correlation between the error rate, the number of classification trees, and the relative importance of 20 genes (Figure 1K, 1L). Additionally, a Venn diagram was used to intersect 40 M6ARGs and DEGs and obtain five crucial m6A regulators (Figure 1M). qRT-PCR analysis revealed that the METTL5 mRNA levels in AS tissues were downregulated when compared with those in healthy tissues (Figure 1N).

### Analysis of immune infiltration

**Figure 2:**
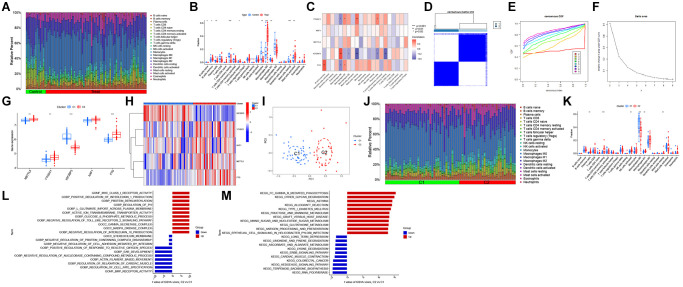
**Identification of N6-methyladenosine (m6A)-related molecular clusters in atherosclerosis (AS).** (**A**) The relative abundances of 22 infiltrating immune cells between AS and non-AS controls. (**B**) Boxplots showing the differential immune cell infiltration levels between AS and non-AS controls. (**C**) Correlation analysis between five m6A regulators and infiltrating immune cells. (**D**) Consensus clustering matrix when k = 2. (**E**) The cumulative distribution function (CDF) curve. (**F**) The relative change in area under the CDF curve for k = 2–9. (**G**, **H**) The differential expression levels of five m6A regulators between two m6A clusters. (**I**) Principal component analysis (PCA) of cluster 1 and cluster 2. (**J**) Boxplots showing the differential immune cell infiltration levels between AS and non-AS controls. (**K**) Differential immune cell infiltration levels between cluster 1 and cluster 2. (**L**) Differentially enriched biological functions between cluster 1 and cluster 2 were ranked based on the t-value obtained using gene set variation analysis (GSVA). (**M**) Differentially enriched Kyoto Encyclopedia of Genes and Genomes (KEGG) pathways between cluster 1 and cluster 2 were ranked based on the t-value obtained using GSVA. ^*^p < 0.05, ^**^p < 0.01, ^***^p < 0.001, ^****^p < 0.0001.

The differential immune features between patients with AS and healthy individuals were examined using CIBERSORT analysis (Figure 2A). The infiltration levels of memory B cells, M0 macrophages, and activated mast cells were upregulated, whereas those of resting memory CD4+ T cells, monocytes, and M1 macrophages were downregulated in AS samples (Figure 2B). Correlation analysis revealed that IGF2BP3 expression was positively correlated with M0 macrophage abundance and negatively correlated with resting mast cell and resting memory CD4+ T cell abundances (Figure 2C). These results indicate that M6ARGs mediate the onset and progression of AS by modulating the infiltration levels of immune cells.

### Identification of m6A clusters in AS

Different m6A clusters associated with AS were identified using a consensus cluster algorithm based on the expression levels of five m6A regulators. When the K value was 2, the consensus algorithm categorized patients with AS into two m6A subtypes (Figure 2D–2F). Compared with those in cluster 1, the expression levels of METTL5, YTHDC1, and FTO were upregulated in cluster 2 (Figure 2G, 2H). PCA (Figure 2I) revealed that the two m6A subtypes exhibited distinct biological processes. CIBERSORT analysis demonstrated that the infiltration levels of plasma cells, resting memory CD4+ T cells, activated natural killer (NK) cells, M1 macrophages, and resting mast cells were upregulated, whereas those of M0 macrophages were downregulated in cluster 1 (Figure 2J, 2K).

### GSVA results

The differential biological activities between the two AS subtypes were examined using GSVA. Cluster 2 was positively correlated with major histocompatibility complex class I receptor activity, positive regulation of interleukin 1 production, and metabolism-related pathways, including glutathione metabolism, amino sugar and nucleotide sugar metabolism, and fructose and mannose metabolism (Figure 2L, 2M).

### GO, KEGG, and DO analysis results

**Figure 3:**
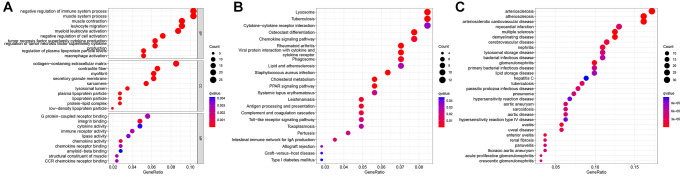
**Functional analysis of differentially expressed genes (DEGs) between two N6-methyladenosine (m6A) subtypes.** (**A**) Gene Ontology (GO) analysis. (**B**) Kyoto Encyclopedia of Genes and Genomes (KEGG) analysis. (**C**) Disease Ontology (DO) analysis.

Functional enrichment analysis revealed that DEGs between the two clusters were enriched in macrophage activation, muscle system processes, and negative regulation of the immune system (Figure 3A). KEGG pathway enrichment analysis demonstrated that DEGs were enriched in the chemokine signaling, PPAR signaling, Toll-like receptor signaling, and lipid and AS pathways (Figure 3B). DO analysis revealed that DEGs were associated with arteriosclerosis, AS, arteriosclerotic heart disease, and aortic aneurysm (Figure 3C).

### Nomogram and ROC curves

**Figure 4:**
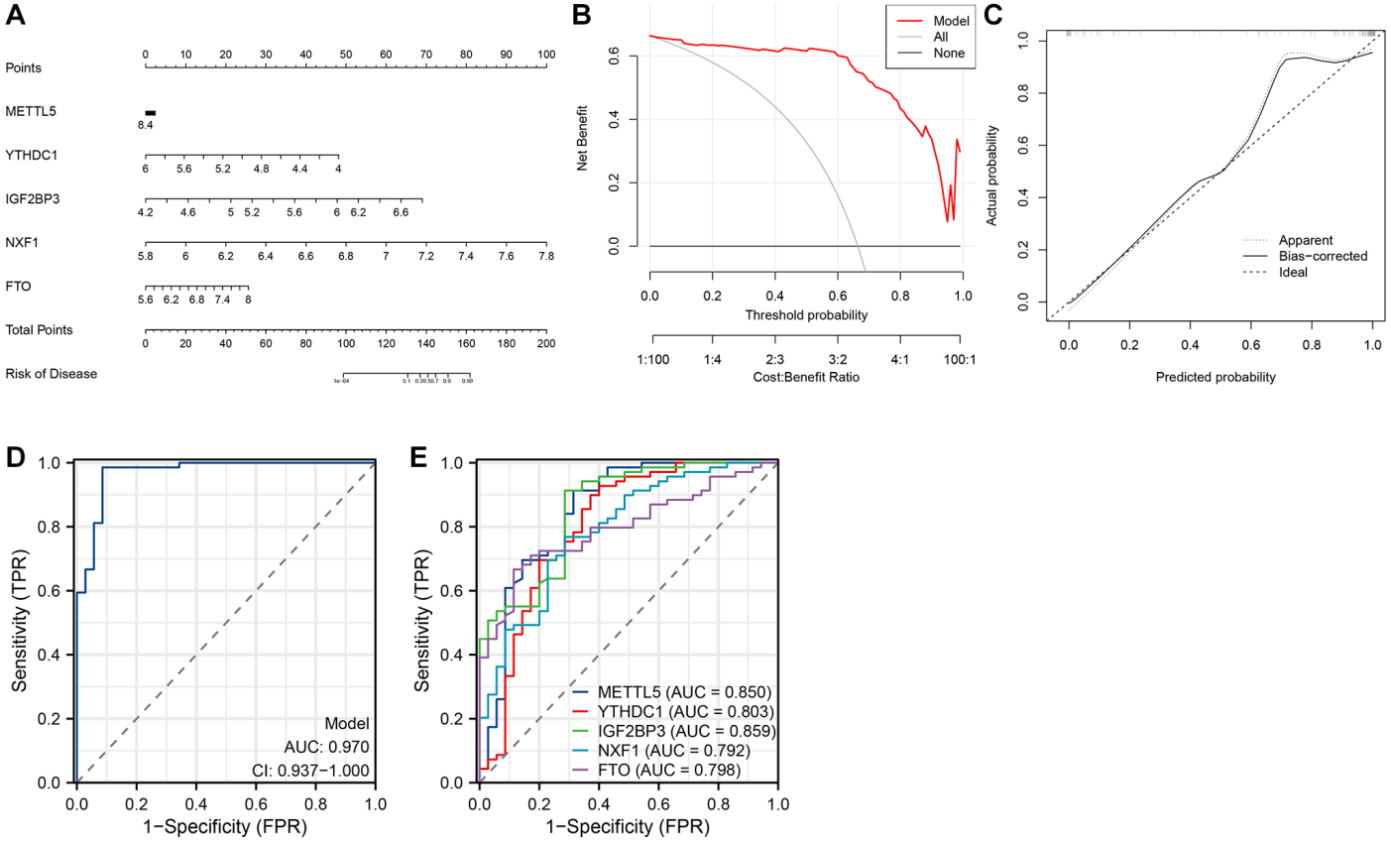
**Establishment of the nomogram model.** (**A**) Establishment of the nomogram model based on five N6-methyladenosine (m6A) regulators. (**B**, **C**) Construction of the calibration curve (**B**) and decision curve analysis (DCA) (**C**) for assessing the predictive efficiency of the nomogram model. (**D**) Receiver operating characteristic (ROC) curves of a five-m6A regulator-based model. (**E**) ROC curves of five m6A regulators.

A nomogram based on M6ARGs was constructed to evaluate the risk of developing AS (Figure 4A). DCA revealed the enhanced predictive ability of the nomogram (Figure 4B). As shown in Figure 4C, the predicted risk was consistent with the actual risk. The five-m6A regulator-based model demonstrated enhanced diagnostic efficacy (Figure 4D). The ROC curve revealed that the area under the curve (AUC) values for YTHDC1, IGF2BP3, NXF1, FTO, and METTL5 were 0.803, 0.859, 0.792, 0.798, and 0.850, respectively (Figure 4E).

### scRNA-seq profiles of AS

**Figure 5:**
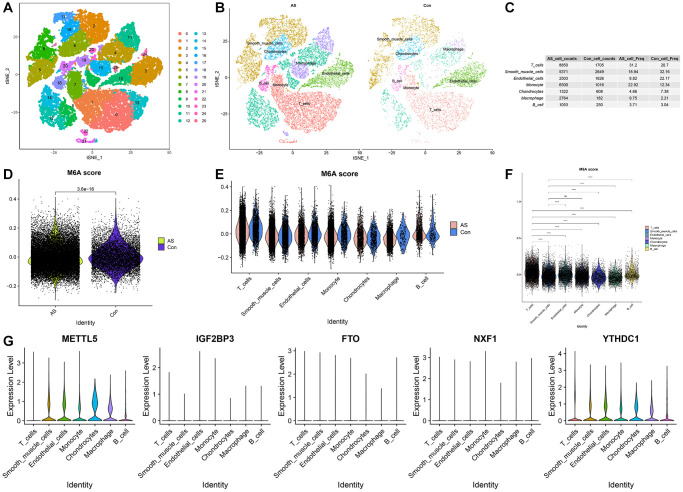
**Cellular composition of the atherosclerosis (AS) tissue microenvironment.** (**A**) The t-stochastic neighbor embedding (t-SNE) plot shows the distribution of 26 major cell subsets. (**B**) Annotation of each cell type in AS and adjacent healthy tissues. (**C**) Proportion of each cell type in AS and healthy tissues. (**D**) The differential N6-methyladenosine (m6A) scores between AS and healthy tissues. (**E**) m6A scores in each cell type. (**F**) The differential m6A scores between cell types. (**G**) Violin plot of the expression levels of five key m6A regulators in each cell type.

After pre-processing the GSE159677 single-cell dataset, the combined data of AS and adjacent healthy tissues were subjected to t-distributed stochastic neighbor embedding (t-SNE) non-linear dimension reduction. The segregation of all cells revealed 26 cell subclusters (Figure 5A). The “FindAllMarkers” function was used to identify DEGs in each cluster (logFC = 0.25). The “singleR” function was used to identify the following seven cell types: B cells, T cells, chondrocytes, smooth muscle cells (SMCs), macrophages, endothelial cells (ECs), and monocytes (Figure 5B). Additionally, the proportions of each cell type in AS and healthy tissues were examined. Compared with those in healthy tissues, the proportions of T cells, monocytes, and macrophages were upregulated and the proportions of SMCs and ECs were downregulated in AS tissues (Figure 5C). The m6A scores were significantly different between AS and healthy tissues (Figure 5D). The levels of m6A regulators in seven cell types are shown in Figure 5E. The m6A regulator levels significantly varied between the seven cell types (Figure 5F). METTL5 was upregulated in specific cell types, including macrophages, SMCs, and ECs (Figure 5G).

## DISCUSSION

AS is a chronic inflammatory disease involving cellular and molecular alterations, such as epigenetic modifications [21–23]. Currently, limited methods are available for the early diagnosis and risk stratification of AS, impeding the development of therapeutic strategies. Hence, there is a need to identify novel diagnostic markers and molecular subtypes to provide useful novel insights for developing clinical interventions for AS. m6A modification is involved in various biological processes in AS [24–26]. Hence, this study aimed to investigate the role of m6A regulators in AS and identify m6A subtypes in AS for risk stratification, precision therapy development, and risk prediction.

This study identified 31 differentially expressed M6ARGs between 35 healthy tissues and 69 AS tissues. WGCNA revealed that the brown module was associated with AS. From the module, 3529 hub genes were extracted using specific filtering criteria. The results of the Venn diagram revealed that five m6A regulators are potential hub genes involved in AS progression. qRT-PCR analysis demonstrated that METTL5 was downregulated in AS tissues, which was consistent with the bioinformatics analysis results. YTHDC1 upregulation is reported to promote neuron survival and ameliorate ischemic brain injury. Mechanistically, YTHDC1 promotes the phosphorylation of AKT1 by degrading PTEN mRNA, resulting in the activation of the anti-apoptotic signaling pathways, including the BCL2 and MTOR pathways [27]. IGF2BP3 enhances reendothelialization after arterial injury by modulating EC proliferation, migration, and apoptosis through the upregulation of VEGFA mRNA stability and activation of the VEGF/PI3K/Akt signaling pathway [28]. FTO inhibits NLRP3-mediated pyroptosis through the suppression of β-catenin ubiquitination and degradation by decreasing the stability of CBL mRNA, contributing to the alleviation of cardiac ischemia/reperfusion injury [29].

The infiltration levels of M0 macrophages, memory B cells, and activated mast cells were upregulated in patients with AS, indicating the important role of immune cell infiltration in AS development. Unsupervised clustering revealed that the infiltration levels of resting memory CD4+ T cells, activated NK cells, and M1 macrophages are upregulated in cluster 2. Enrichment analysis confirmed that the DEGs between two m6A subtypes are involved in activating macrophages and immune-related pathways, such as the chemokine signaling, Toll-like receptor signaling, and antigen processing and presentation pathways. CCL8 enhances the permeability of ECs and downregulates the levels of TJP1 and CDH5, impairing the function of the endothelial barrier and facilitating AS development [30]. Mechanistically, CCL8 activates the PI3K/AKT, ERK1/2, and NF-κB signaling cascades by binding to CCR1 and CCR2, regulating the generation of NOX2-induced reactive oxygen species. Therefore, m6A modification mediates AS progression by regulating the immune microenvironment through the modulation of the immune-associated pathways.

Single-cell analysis revealed that, compared with those in healthy tissues, the infiltration levels of T cells, monocytes, and macrophages were upregulated and the infiltration levels of SMCs and ECs were downregulated in AS tissues. DCLK1 is upregulated in the infiltrating macrophages of AS tissues, promoting inflammatory responses and plaque formation. Previous studies have reported that DCLK1 promotes IKBKB phosphorylation through direct binding, activating the NF-κB signaling pathway and regulating the expression of inflammatory genes [31]. Macrophage lipid accumulation and uptake can be inhibited by USP9X. The downregulation of USP9X in macrophages promotes the formation of foam cells and inflammatory responses, contributing to the progression of AS. USP9X removes K63 polyubiquitination at the K27 site of the class A1 scavenger receptor (SR-A1). The deubiquitination process impairs the internalization of SR-A1 after its interaction with oxidized low-density lipoprotein (ox-LDL), decreasing the uptake of ox-LDL in macrophages [32]. The m6A score was significantly different between AS and healthy tissues, suggesting that m6A methylation may be involved in AS progression. However, METTL5 was upregulated in macrophages, SMCs, and ECs. Therefore, this study hypothesized that these cell types may modulate m6A methylation levels through the regulation of METTL5 expression, promoting the onset and development of AS. The nomogram model exhibited an enhanced predictive power for assessing the risk of developing different AS subtypes. ROC analysis revealed that the m6A regulators with AUC values > 0.8 were METTL5 (0.850), YTHDC1 (0.803), and IGF2BP3 (0.859).

This study systematically analyzed the expression of m6A regulators in AS and evaluated the potential of five m6A regulators to serve as diagnostic markers for AS. However, this study has some limitations. This study did not examine the correlation between METTL5 expression and immune cell infiltration. Additionally, the molecular mechanisms of METTL5 in the progression of AS were not elucidated.

## CONCLUSIONS

A five-m6A regulator-based model was used to predict the risk of developing different AS subtypes. The levels of METTL5 were upregulated in macrophages, SMCs, and ECs. Thus, these cell types may promote AS progression through the modulation of m6A methylation levels by regulating METTL5 expression.
